# Impact of Neoadjuvant Chemotherapy on Survival Outcomes in Gastric Signet-Ring Cell Carcinoma

**DOI:** 10.3390/cancers17142400

**Published:** 2025-07-19

**Authors:** Salvatore Sorrenti, Silvia Malerba, Eleonora Lori, Daniele Pironi, Karol Polom, Jaroslaw Skokowski, Sergii Girnyi, Tomasz Cwalinski, Francesco Paolo Prete, Yogesh K. Vashist, Mario Testini, Luigi Marano

**Affiliations:** 1 Department of Surgery, “Sapienza” University of Rome, 00161 Roma, Italy; salvatore.sorrenti@uniroma1.it (S.S.); eleonora.lori@uniroma1.it (E.L.); daniele.pironi@uniroma1.it (D.P.); 2 Department of Precision and Regenerative Medicine and Ionian Area, University of Bari “Aldo Moro”, 70110 Bari, Italy; s.malerba10@studenti.uniba.it (S.M.); francesco.prete@uniba.it (F.P.P.); mario.testini@uniba.it (M.T.); 3 Department of Medicine, Academy of Applied Medical and Social Sciences—AMiSNS (Akademia Medycznych I Spolecznych Nauk Stosowanych), 52-300 Elbląg, Poland; k.polom@amisns.edu.pl (K.P.); j.skokowski@amisns.edu.pl (J.S.); yogesh.vashist@outlook.de (Y.K.V.); 4 Department of General Surgery and Surgical Oncology, “Saint Wojciech” Hospital, “Nicolaus Copernicus” Health Center, 80-000 Gdańsk, Poland; sgirnyi@copernicus.gda.pl (S.G.); tcwalinski@copernicus.gda.pl (T.C.); 5 Department of Gastrointestinal Surgical Oncology, Greater Poland Cancer Centre, Garbary 15, 61-866 Poznan, Poland; 6 Department of Surgical Oncology, Asklepios Harz Clinic Goslar, 38640 Goslar, Germany

**Keywords:** gastric signet-ring cell carcinoma, neoadjuvant therapy, survival outcomes, subgroup analysis, tumor location, population-based cohort

## Abstract

**Background**: Gastric signet-ring cell carcinoma (GSRCC) is an aggressive gastric cancer subtype with limited evidence supporting the role of neoadjuvant chemotherapy (NAC). This study evaluated the impact of NAC on overall survival (OS) in a large, population-based cohort. **Methods**: We analyzed data from the SEER database (2011–2018), identifying patients aged 20–80 years with primary gastric tumors (C16.0–C16.9) and signet-ring cell histology who underwent curative-intent gastrectomy. Patients with metastatic disease, non-curative surgery, clinical Stage I, incomplete staging, or unknown survival were excluded. OS was assessed using Kaplan–Meier analysis and multivariable Cox regression. Subgroup analyses evaluated the interaction of NAC with tumor location and clinical stage. **Results**: A total of 978 patients met inclusion criteria; 436 (44.6%) received NAC. The 3- and 5-year OS rates were 43.9% and 38.3%, respectively. NAC was not associated with improved OS compared to surgery alone (5-year OS: 39.7% vs. 37.2%; log-rank *p* = 0.34) and was not an independent prognostic factor in multivariable analysis (*p* = 0.651). Independent predictors of worse OS included larger tumor size, advanced stage, positive nodal status, and Black race (all *p* < 0.05). Subgroup analysis indicated a survival benefit from NAC in patients with mid or distal gastric tumors (*p* < 0.001 for interaction). **Conclusions**: In this SEER-based analysis, NAC did not improve OS in the overall GSRCC population. However, selected subgroups may derive benefit, supporting a personalized approach to neoadjuvant therapy in GSRCC.

## Simple Summary

Gastric signet-ring cell carcinoma is a distinct and aggressive histologic subtype of stomach cancer, which typically presents at an advanced stage and is associated with poor prognosis. While neoadjuvant chemotherapy is commonly employed in the treatment of gastric cancer, its benefit in signet-ring cell carcinoma remains uncertain. In this population-based study, we analyzed data from a large United States cancer registry to evaluate whether patients with this tumor type experienced improved survival when treated with neoadjuvant chemotherapy. Our findings indicate that, in the overall population, neoadjuvant chemotherapy did not confer a long-term survival advantage compared to surgery alone. However, selected subgroups—particularly those with tumors located in the middle or lower stomach, or with more advanced disease—appeared to derive benefit. These results highlight the importance of a more individualized treatment approach and may inform future research and clinical decision-making in this challenging gastric cancer subtype.

## 1. Introduction

Gastric cancer is the fifth most common malignancy worldwide, with over one million new cases annually [1]. Among its histological subtypes, gastric signet-ring cell carcinoma (GSRCC) is characterized by diffusely infiltrative growth, with >90% of cells containing intracytoplasmic mucin that displaces the nucleus. GSRCC accounts for approximately 14–35% of gastric cancers globally [2]. Clinically, GSRCC behaves aggressively, often presenting at advanced stages; up to 60–80% of patients have locoregionally advanced disease at diagnosis [3]. Consequently, GSRCC portends a worse prognosis than non-signet adenocarcinoma, with 5-year survival rates for locally advanced cases under 20% [4]. This dismal outcome has prompted investigation into multimodal treatment approaches. Neoadjuvant chemotherapy (NAC) is an established component of therapy for locally advanced gastric adenocarcinoma in many guidelines, based on trials like MAGIC and FLOT4 that demonstrated survival benefits with perioperative chemotherapy in mixed histology gastric cancers [5,6]. However, the efficacy of NAC specifically for GSRCC remains controversial. Major clinical trials have seldom included subtype-specific analyses for signet-ring histology. For instance, the MAGIC trial, which set a perioperative chemotherapy standard in Europe, did not report outcomes separately for GSRCC, leaving uncertainty with regard to this subgroup [6]. Some evidence even suggests that NAC might be less effective or detrimental in GSRCC. A French multicenter study of 1050 patients reported worse survival with perioperative chemotherapy vs. surgery alone in the signet-ring subset (hazard ratio [HR]= 1.40, 95% confidence intervals (95% CIs): 1.1–1.9,) [7]. Similarly, several retrospective series have found no clear survival benefit of NAC in GSRCC [8,9,10]. A meta-analysis focusing on esophagogastric signet-ring cancers showed no significant improvement in survival with NAC (pooled HR = 1.01, *p* = 0.98) [11]. In contrast, other studies suggest a possible advantage: for example, a Dutch nationwide registry (*n* = 2046 diffuse-type tumors) noted significantly reduced 90-day postoperative mortality with NAC (HR= 0.29, 95% CI 0.20–0.44) [9], and an analysis by Heger et al. observed prolonged median OS with NAC in signet-ring cases (28.5 vs. 14.9 months, *p* < 0.001) [12]. Given these conflicting data, current clinical guidelines offer only cautious recommendations for NAC in diffuse-type or signet-ring gastric cancer. The European Society for Medical Oncology (ESMO) suggests considering NAC for diffuse gastric cancers but emphasizes the lack of high-level evidence specific to GSRCC. A dedicated phase III trial, PRODIGE 19 (NCT01717924), was initiated to compare perioperative versus adjuvant chemotherapy in GSRCC, but the results are pending [13]. Thus, there remains a critical need for robust, GSRCC-focused research to guide therapy. Here, we present an analysis of NAC in GSRCC using a large population-based Western cohort from the SEER database.

This study provides, to our knowledge, one of the largest samples of GSRCC patients treated with and without NAC in a Western population. By focusing on the SEER cohort alone, we aim to clarify the impact of NAC on survival outcomes in an unselected, real-world GSRCC population and to identify subgroups that might derive benefit. Our findings seek to inform clinical decision-making and future trial design for this challenging histological subtype.

## 2. Materials and Methods

### 2.1. Study Population

**Figure 1:**
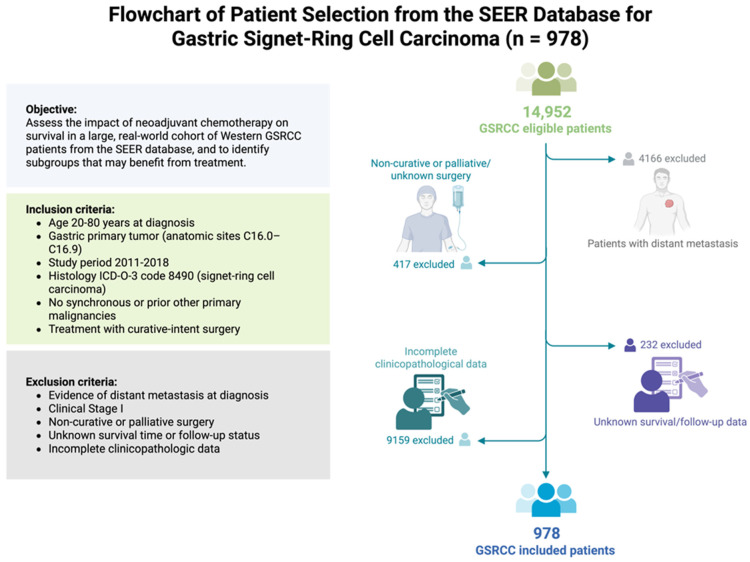
Flowchart of patient selection from the SEER database for gastric signet-ring cell carcinoma (GSRCC) (*n* = 978).

We performed a retrospective cohort study using data from the Surveillance, Epidemiology, and End Results (SEERs) database (SEER*Stat v8.4.2. Bethesda, MD, USA) to identify patients with gastric signet-ring cell carcinoma diagnosed from January 2011 through December 2018 [14]. The inclusion criteria were as follows: (1) gastric primary tumor (anatomic sites C16.0–C16.9); (2) histology ICD-O-3 code 8490 (signet-ring cell carcinoma); (3) aged 20–80 years at diagnosis; (4) no synchronous or prior other primary malignancies; and (5) surgical treatment with curative intent (gastrectomy). The exclusion criteria were as follows: (1) evidence of distant metastasis at diagnosis (SEER historic stage distant); (2) non-curative or palliative surgery (including local tumor destruction only, or no cancer-directed surgery); (3) unknown survival time or follow-up status; and (4) incomplete clinicopathologic data such as undefined tumor stage. We also excluded cases with clinical stage I disease (T1N0, T1N1, T2N0). Additionally, cases with missing T or N classification were excluded, given that accurate pathologic staging is essential for robust outcome analysis. The selection process is outlined in the study flow diagram (Figure 1). This study utilized de-identified data from the public SEER database; therefore, institutional review board approval and informed consent were not required. The analysis conforms to the Declaration of Helsinki and all relevant guidelines for research on human datasets.

### 2.2. Treatment Definitions

For this analysis, patients were categorized into two treatment groups. The surgery-alone group included patients who underwent surgical resection without any documented neoadjuvant therapy. Although some of these patients received adjuvant therapy after surgery in accordance with standard indications, no preoperative chemotherapy was administered. The NAC group consisted of patients who received neoadjuvant chemotherapy prior to surgery. In the SEER database, neoadjuvant treatment was inferred from records of chemotherapy administration and its timing relative to surgery, when available. Patients who received preoperative chemotherapy were included in this group regardless of whether they also received postoperative chemotherapy. All patients in the NAC group subsequently underwent gastrectomy. Detailed information on the extent of surgery (such as total versus subtotal gastrectomy) and the scope of lymphadenectomy is limited in SEER; thus, surgical quality indicators such as D2 dissection are not explicitly recorded. We assumed that all included patients underwent at least a standard gastrectomy with lymph node removal, consistent with curative intent. Adjuvant therapy, defined as chemotherapy and/or radiotherapy administered postoperatively, was documented based on SEER treatment fields and included in the analysis as a separate covariate.

### 2.3. Outcome and Follow-Up

The primary endpoint was overall survival (OS), defined as the time from the initiation of definitive treatment (surgery for the surgery-alone group, or start of NAC for the NAC group) to death from any cause. Patients alive at the last follow-up were censored. SEER provides survival time in months and vital status, which we used to calculate OS. We focused on 3-year and 5-year OS rates as key metrics. Given the retrospective nature, treatment allocation was not randomized; thus, baseline characteristics and potential confounders were compared between groups. Patients were followed by SEER participating institutions according to standard postoperative surveillance protocols, which typically include periodic clinical exams and imaging. The SEER database captures survival status via linkage to national death indices and follow-up from reporting hospitals. In this study, follow-up was available through the end of 2018 (with some cases updated in 2019). The median follow-up duration was calculated using reverse Kaplan–Meier methodology [15].

### 2.4. Statistical Analysis

Descriptive statistics were used to summarize patient demographics, tumor characteristics, and treatments. Continuous variables were expressed as the mean ± standard deviation (SD) or median (interquartile range, IQR) as appropriate; categorical variables were reported as frequencies and percentages. Differences in characteristics between groups (NAC vs. surgery alone) were evaluated with Chi-square or Fisher’s exact tests for categorical variables and Student’s *t*-test for continuous variables. OS was estimated using the Kaplan–Meier method. The log-rank test was applied to compare survival curves between groups (NAC vs. no NAC) in the overall cohort and within subgroups. We reported 3-year and 5-year OS rates. Cox proportional hazards regression was employed to identify independent prognostic factors for OS. Candidate covariates included treatment group (NAC vs. surgery alone), age, sex, race, year of diagnosis, tumor location, tumor size, clinical Tumor Node Metastasis (TNM) stage (cTNM), pathologic T stage (pT), pathologic N stage (pN), tumor differentiation grade, and use of adjuvant therapy. All covariates were entered into the initial Cox model. A stepwise backward elimination was then used, removing factors with *p* > 0.10, to arrive at a parsimonious model; however, factors of known clinical importance were retained regardless of significance to control for confounding (notably age and sex were kept in the final model). Hazard ratios (HRs) with 95% confidence intervals (CIs) and *p*-values were calculated. Proportional hazards assumptions were checked using Schoenfeld residuals and found to be satisfied. We performed exploratory subgroup analyses to examine the effect of NAC on OS within specific strata: (1) tumor location (proximal vs. mid vs. distal stomach) and (2) clinical stage (II vs. III). These subgroups were chosen a priori based on clinical interest and a sufficient sample size. Interaction tests were conducted by adding an interaction term between treatment and subgroup variable in the Cox model to assess whether the impact of NAC differed by subgroup. To reduce the risk of overfitting, the initial multivariable Cox regression model included clinically and biologically relevant variables selected a priori based on existing literature and expert knowledge, and backward elimination was applied only to remove non-contributory variables while preserving the interpretability and robustness of the model. A two-tailed *p* < 0.05 was considered statistically significant for all analyses. All statistical analyses were performed using IBM SPSS Statistics for Windows, Version 26.0 (IBM Corp., Armonk, NY, USA).

## 3. Results

### 3.1. Patient Characteristics

After applying all exclusion criteria, a total of 978 patients diagnosed with gastric signet-ring cell carcinoma were included in the final analysis from the SEER database (Figure 1).

The baseline characteristics of these patients are summarized in Table 1. The median age was 61 years (range 22–79), and there was a slight male predominance (males comprised 52.9% of the cohort; females comprised 47.1%). Most patients were White (65.4%), followed by Black patients (13.3%) and Asian/Pacific Islander patients (3.8%). Tumors were predominantly located in the distal stomach (29.6%), followed by the mid (28.4%) and proximal (17.8%) stomach, while the location was unknown in 14.8% of cases. The tumor size was ≤5 cm in 46.0% of cases, >5 cm in 25.4%, and not recorded in 28.6%. Over half of the patients (51.4%) were diagnosed in the earlier part of the study period (2011–2014). Notably, 100% of patients included in the final cohort presented with locally advanced disease (clinical stage II–III) at diagnosis, as patients with clinical stage I or unknown stage were excluded by study design. Specifically, 20.6% had clinical stage II and 79.4% had stage III disease. This stage distribution underscores the advanced presentation typical of GSRCC. All patients included had no distant metastases, in accordance with the inclusion criteria.

Treatment details are provided in Table 2. Out of 978 patients, 436 patients (44.6%) received neoadjuvant chemotherapy (NAC) prior to surgery, reflecting the real-world reluctance or limited indication toward using NAC for GSRCC during the study period. The remaining 542 patients (55.4%) underwent surgery first without NAC. Among those who did not undergo NAC, some received adjuvant therapy after surgery as per clinical judgment. In the entire cohort, 41.6% of patients received some form of postoperative treatment: 16.7% had adjuvant chemotherapy alone, 21.9% received combined chemoradiotherapy, and 3.0% had adjuvant radiotherapy only. However, the majority (58.4%) did not receive any adjuvant therapy (often due to patient factors, or perhaps rapid recurrence or death before planned therapy). Importantly, the data highlight that upfront surgery remained the predominant strategy for GSRCC in the United States during 2011–2018, with NAC reserved for a subset (roughly one in four patients). This likely reflects strict selection of NAC for those with more advanced local disease or as part of clinical trial protocols. SEER does not report the specific chemotherapeutic regimens; at the time, common NAC regimens would have included platinum/5-FU combinations (e.g., FOLFOX, ECF) and, later, FLOT, as supported by contemporaneous trials [5]. Details of the surgical approach (total vs. subtotal gastrectomy, lymph node dissection extent) are not captured in SEER; however, all patients had a gastrectomy with curative intent. The mean number of lymph nodes examined pathologically in the resection specimen was 27.2 (±39.7), which suggests that many patients had extensive nodal evaluation (this high standard deviation indicates a wide range, with some cases showing extremely high counts, possibly due to multiple procedures or differing pathology practices). The mean number of metastatic lymph nodes was 18.7 (±23.4), indicating a high nodal tumor burden on average, consistent with advanced disease.

### 3.2. Histopathological Findings

Pathologic examination of resected specimens is summarized in Table 3. Given the inclusion criteria, all tumors were stage M0. The pathological T stage distribution in SEER patients was as follows: T1 in 28.3%, T2 in 16.8%, T3 in 27.7%, and T4 in 27.2%. Thus, the majority (54.9%) had tumors invading beyond the muscularis propria (T3–T4) at resection, despite many being referred to as stage II/III clinically (some downstaging may have occurred with NAC in certain cases). For pathological nodal status, 40.4% had node-negative disease (pN0). Positive lymph nodes were present in about half of the patients: pN1 in 24.1%, pN2 in 18.9%, and pN3 in 16.6%. The high proportion of pN3 (metastasis in ≥7 nodes) again reflects the advanced nature of many GSRCCs. Cases with unknown nodal status were excluded from the final analysis, suggesting that all patients underwent lymph node evaluation. The median number of positive nodes was substantial, contributing to the poor prognosis. All tumors were of poor histological differentiation. No cases of well-, moderately-, or undifferentiated tumors were included in the revised cohort, as G1, G2, and G4 grades were excluded for consistency with histological criteria for SRCC. These findings underscore that by the time of surgical resection, GSRCC typically exhibits high-grade features. The lymph node yields have been noted above (mean 27 nodes examined). While SEER does not directly state whether a D1 or D2 lymphadenectomy was performed, the average nodal count suggests many patients had an adequate dissection (often >15 nodes, as recommended [16]). The mean number of positive nodes was 18.7, which is high; it is likely that a significant subset of patients had extensive nodal involvement (consistent with the pN3 category). This highlights the aggressive nodal spread propensity of signet-ring tumors.

### 3.3. Survival Outcomes

**Figure 2:**
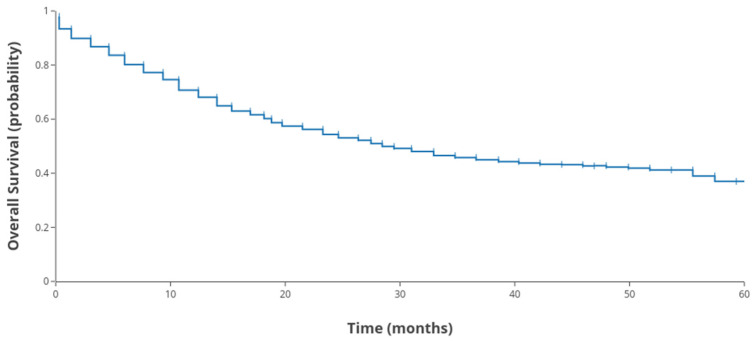
Kaplan–Meier survival curve for the whole SEER Cohort (*n* = 978).

The median follow-up time for the revised cohort of 978 patients was 30 months (range: 8–131 months). Given the aggressive biological behavior of gastric signet-ring cell carcinoma and the enrichment of clinically advanced cases, overall survival outcomes remained poor. By the end of the study period, a substantial proportion of patients had died. The 3-year overall survival (OS) rate was 43.9%, and the 5-year OS rate was 38.2%. The Kaplan–Meier survival curve for the entire SEER cohort (regardless of treatment group) is depicted in Figure 2. Early mortality within the first year after surgery was substantial, and the survival curve plateaued at a low level by 5 years.

**Figure 3:**
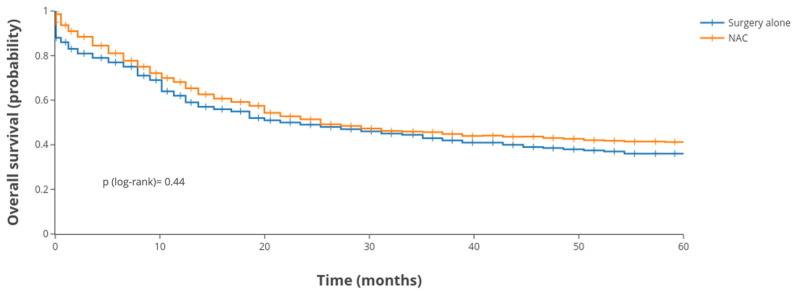
Overall survival: NAC vs. surgery in SEER cohort. NAC—neoadjuvant chemotherapy.

When comparing NAC vs. surgery alone, no significant difference in long-term survival was observed in the overall analysis. The Kaplan–Meier survival curves stratified by treatment group (NAC vs. no NAC) are presented in Figure 3. Patients who received NAC had a 5-year OS of 39.7%, compared to 37.2% in those who underwent surgery without NAC (log-rank *p* = 0.44). During the first 2–3 years, there was no apparent survival advantage conferred by NAC. By 5 years, the slight numerical difference favored the NAC group, but this was not significant. This result held true even when adjusting for other factors (see the multivariable results below). Thus, NAC did not improve overall survival in unselected GSRCC patients in this cohort.

To further explore outcomes, we analyzed prognostic factors affecting OS. Univariate analysis (Table 4) identified several factors associated with worse survival: Asian race (compared to Black race), a larger tumor size (>5 cm), a higher clinical stage, advanced pathological T and N stages, and lack of adjuvant therapy were all linked to inferior OS (*p* < 0.05 for each). Neither patient sex nor age at diagnosis had a significant impact on OS in univariate analysis of the SEER cohort. The hazard ratio for patients aged >60 was <1 (though not significant), suggesting younger patients might have had slightly worse outcomes, potentially reflecting the aggressive biology in some younger cases, but this did not reach significance. The year of surgery (2011–2014 vs. 2015–2018) was also not a significant predictor, indicating no strong period effect or improvement in outcomes over the studied timeframe.

We then performed a multivariable Cox regression including key covariates. After adjustment, the factors that emerged as independent predictors of poorer OS were race, tumor size, clinical stage, pathologic T stage, and pathologic N stage (all *p* < 0.05) in the SEER cohort. Specifically, Black patients had worse survival compared to White and Asian patients even after controlling for other factors, suggesting possible disparities or tumor biology differences (hazard ratio for White vs. Black = 0.95, for Asian vs. Black = 0.58; see Table 4, *p* = 0.695 and *p* = 0.033, respectively). Tumor size >5 cm remained independently associated with higher mortality (adjusted HR = 1.67 vs. tumors ≤5 cm, *p* < 0.001), indicating that bulky tumors portend worse outcomes. Advanced clinical stage at presentation continued to influence survival: patients with cStage III had a significantly higher hazard of death than those with cStage II (adjusted HR = 2.98, *p* = 0.001). Similarly, a higher pathologic T category (especially T3, T4) and nodal status (pN1 and pN3) were strong independent predictors of poor OS. Specifically, pathologic T3 had an HR of 1.52 (*p* = 0.017), T4 an HR of 2.13 (*p* < 0.001), pN1 an HR of 1.34 (*p* = 0.035), and pN3 had an HR of 1.64 (*p* = 0.002). These findings underscore that the extent of disease, both preoperatively assessed and pathologically confirmed, drives survival in GSRCC. To mitigate confounding due to adjuvant treatment, we included postoperative therapy status as a covariate in the multivariable Cox regression model. This adjustment aimed to partially account for the influence of post-surgical treatment and better isolate the effect of NAC on overall survival. Despite this adjustment, NAC was not found to be independently associated with improved survival outcomes.

Crucially, NAC was not an independent predictor of OS on multivariable analysis, which was consistent with the unadjusted findings. The hazard of death for patients who did not receive NAC was essentially the same as for those who did (adjusted HR = 0.91, 95% CI 0.74–1.13, *p* = 0.397; reference: NAC group), implying no significant survival benefit or detriment associated with NAC after accounting for stage, size, etc. In other words, once we adjust for the fact that NAC patients tended to have more advanced disease, NAC itself did not significantly alter the risk of mortality. Adjuvant therapy also did not show a clear independent effect on OS in this cohort (patients who received adjuvant chemo ± radiotherapy (RT) did not have significantly different adjusted survival compared to those who did not; HR = 1.17, *p* = 0.285), possibly due to indication bias in which only higher-risk patients received adjuvant therapy.

In multivariable analysis (Table 4), race remained significant: Asian patients had about half the risk of death compared to Black patients (HR = 0.58, *p* = 0.033). White patients had a trend toward better survival than Black patients (HR = 0.95), but this did not reach significance (*p* = 0.695). A tumor size of >5 cm conferred roughly 1.6 times the hazard of ≤5 cm tumors (*p* < 0.001). Clinical stage III at presentation had nearly threefold the mortality risk of stage II (HR 2.98, *p* = 0.001), underscoring the prognostic value of initial staging even after surgery. Pathologic T3 and T4 were associated with ~1.5-fold and 2.1-fold higher hazards, respectively, than T2 (T3: HR 1.52, *p* = 0.017; T4: HR 2.13, *p* < 0.001). Pathologic N1 and N3 categories were also independently prognostic (N1: HR 1.34, *p* = 0.035; N3: HR 1.64, *p* = 0.002). Notably, clinical stage II and pathologic N2 did not remain significant after adjustment (N2: HR = 1.25, *p* = 0.197), possibly because of interaction or collinearity effects. Year of surgery and patient sex/age were not independent predictors in the final model.

After adjusting for all the above factors, NAC was still not associated with a statistically significant improvement in OS (adjusted HR = 0.91, *p* = 0.397 for no NAC vs. NAC). This reinforces that, in this broad unselected cohort, NAC did not significantly prolong survival compared to surgery-first treatment. To ensure this was not due to an imbalance in adjuvant therapy, we also checked models stratifying by adjuvant treatment and found no significant NAC benefit in either stratum.

### 3.4. Subgroup Analyses

Given the overall negative result for NAC, we explored whether specific subgroups of patients might derive benefit from NAC [7,9,10,11,13,16].

**Figure 4:**
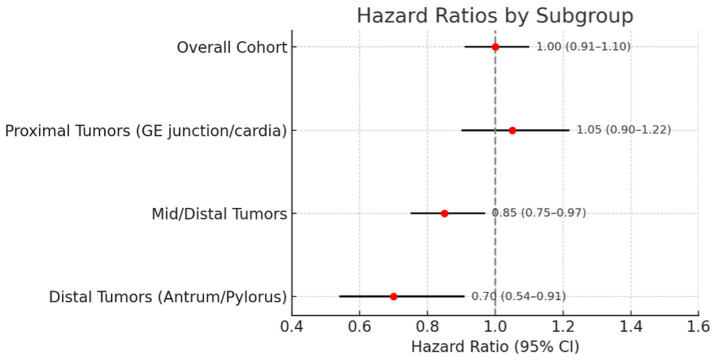
Forest plot: effect of neoadjuvant chemotherapy by tumor location and clinical stage. Forest plot illustrating the effect of neoadjuvant chemotherapy (NAC) versus upfront surgery on overall survival in gastric signet-ring cell carcinoma (GSRCC), stratified by tumor location. NAC was associated with a survival benefit in patients with mid or distal gastric tumors, with hazard ratios below 1.0. In contrast, no benefit from NAC was observed in proximal tumors, where the hazard ratio approached or exceeded 1.0. These exploratory subgroup analyses suggest potential context-specific efficacy of NAC in GSRCC and support a more individualized approach to its application. Hazard ratios (HRs) and 95% confidence intervals (CIs) are shown.

Patients were stratified by tumor location into proximal (GE junction/cardia) vs. mid/distal stomach, because diffuse cancers in the proximal stomach are sometimes thought to behave differently (and proximal tumors might be less responsive to systemic therapy, as they can present as linitis plastica of the cardia). We found a notable difference: in the subset of patients with mid or distal gastric tumors, NAC was associated with improved survival, whereas in proximal tumor patients, NAC showed no benefit. Specifically, interaction testing between treatment and location was significant (*p* < 0.001), indicating the effect of NAC on OS differed by tumor site. Figure 4 illustrates this interaction: the hazard ratio for NAC (vs. no NAC) was <1 in the mid/distal group, suggesting a benefit, whereas it was ≥1 for proximal tumors. We further examined distal stomach tumors (antrum/pylorus) as an isolated subgroup: Kaplan–Meier analysis revealed that NAC significantly improved 3-year OS in distal tumor patients (3-year OS 50% with NAC vs. 30% with surgery alone, *p* = 0.0097). At 5 years, the survival advantage with NAC in distal tumors persisted, although absolute numbers were small. For mid-stomach tumors, a positive trend was also observed (with the NAC group faring better), though numbers were more limited when split out and significance was borderline. In contrast, for proximal tumor patients, there was no hint of benefit from NAC; if anything, outcomes were slightly better in the surgery-only group, though not significantly. This suggests NAC may be more effective for tumors in the body/antrum of the stomach than for those in the gastric cardia. It is important to note that these findings are exploratory and hypothesis-generating; they warrant validation in prospective studies before informing clinical decision-making.

## 4. Discussion

Gastric signet-ring cell carcinoma poses a significant therapeutic challenge due to its aggressive nature and limited response to chemotherapy [16]. In this population-based study of 978 patients from the SEER registry, we found NAC did not significantly improve overall survival in the overall cohort. This finding aligns with prior evidence but also suggests potential benefit in selected subgroups, particularly patients with mid- or distal gastric tumors and those with clinically advanced disease. These observations reinforce the heterogeneity of GSRCC and the need for individualized treatment strategies.

Our findings corroborate earlier retrospective studies and meta-analyses. Messager et al. reported worse survival in SRCC with perioperative chemotherapy [7], while Schiefer et al. found no significant OS advantage with NAC in diffuse-type cancers (HR = 1.0, *p* = 0.98) [17]. Similarly, in our cohort, 5-year OS was 39.7% for NAC patients versus 37.2% for those who underwent surgery without NAC. Notably, it is important to interpret comparisons with Asian datasets in the context of fundamental differences in gastric cancer epidemiology, staging, and treatment paradigms. In countries such as Japan and South Korea, population-level screening programs contribute to a substantially higher proportion of early-stage diagnoses, which significantly inflates reported 5-year survival rates—often exceeding 70% [2,18]. Furthermore, differences in staging classifications, surgical standards (e.g., routine D2 lymphadenectomy), and a greater reliance on adjuvant rather than perioperative chemotherapy further limit direct comparability with Western cohorts. These disparities highlight the need for region-specific treatment strategies and reinforce that higher survival in Eastern populations likely reflects earlier detection and differing healthcare infrastructures, rather than the superior oncologic efficacy of neoadjuvant approaches [19].

A novel and relevant finding is the differential impact of NAC by tumor location. Our subgroup analysis revealed a survival advantage with NAC in mid/distal gastric tumors, while no benefit was evident in proximal tumors. This may relate to variations in tumor biology and drug accessibility. Proximal GSRCC often exhibits diffuse infiltration (linitis plastica) and may harbor features like RHOA mutations and chromosomal instability, which can limit chemotherapeutic efficacy [2,20,21]. In contrast, mid/distal tumors more frequently display CDH1 mutations and microsatellite-stable/genomically stable phenotypes, which may confer greater platinum sensitivity [22]. Vascular architecture and lymphatic drainage may also enhance drug delivery in distal sites. These biologically plausible explanations support tailoring NAC decisions based on tumor location.

These results align with previous reports suggesting anatomical differences in chemosensitivity among GSRCC cases. Heger et al. previously demonstrated improved survival with NAC in advanced signet-ring tumors [12], likely due to downstaging and improved resection margins [23]. While we could not assess post-treatment staging due to SEER limitations, prior evidence suggests NAC may reduce pathologic tumor burden. Nonetheless, this benefit appears insufficient to influence OS broadly, likely due to early systemic dissemination in GSRCC.

One likely explanation for the limited OS gain is intrinsic chemoresistance. GSRCC is characterized by discohesive tumor cells, dense stroma, mucin production, and hypoxic niches—factors that impair drug penetration and response [11,16,18,21,24]. Additionally, most GSRCCs fall under the genomically stable TCGA subtype, which responds poorly to standard regimens [22]. Lack of HER2 amplification and high expression of efflux transporters further diminish the efficacy of chemotherapy. Hence, tumor biology may outweigh any advantage conferred by treatment intensification.

Another limitation pertains to SEER’s treatment data granularity. NAC was classified using available coding on chemotherapy timing, but SEER does not specify regimens, cycles, or completion rates. Consequently, some patients may have received incomplete NAC or chemoradiation. Similarly, surgery-alone patients may have undergone adjuvant therapy. We attempted to mitigate this by including adjuvant therapy as a covariate in multivariable models, but residual heterogeneity cannot be excluded. Future prospective registries with granular treatment data are needed to clarify NAC’s true impact.

Patient selection also warrants consideration. NAC recipients may have been younger or fitter, and although we adjusted for several covariates—including age, race, clinical stage, tumor location, and nodal status—unmeasured confounding remains possible. We considered propensity score matching but opted for multivariable regression given SEER’s moderate sample size and limited covariate availability. Importantly, despite potential selection bias in favor of NAC, we still observed no overall survival benefit, strengthening the argument that GSRCC may not be broadly responsive to NAC.

Our prior investigations have also highlighted the prognostic relevance of SRC percentage. We previously proposed a >10% SRC threshold as more biologically meaningful than the >90% cut-off suggested by IGCA [4,25,26]. However, SEER lacks histologic quantification, and thus the GSRCC cohort likely included both pure and mixed subtypes. This limitation hinders stratification by SRC burden and interpretation of chemosensitivity.

Nonetheless, we found that patients with >10% SRC had superior outcomes compared to those with lower SRC content (5-year OS: 51.8% vs. 15.3%, *p* < 0.001) [4,25]. This contradicts older assumptions that SRC presence uniformly indicates poor prognosis and suggests that a high SRC burden may define a distinct, less aggressive phenotype. Given prior studies demonstrating differential chemosensitivity (e.g., greater response to FLOT in SRC tumors [5]), SRC content should be considered a factor in future trials and treatment stratification.

This study has several strengths: it utilizes a large, nationally representative database and focuses on a histologically defined, clinically challenging subset. Our multivariable analysis and subgroup evaluations offer insights into where NAC may be appropriate. By restricting analysis to Western SEER data, we minimize geographic confounding and improve the relevance for Western oncologic practice.

However, limitations remain. SEER lacks information on margin status (R0/R1), chemotherapy completion, response, and perioperative morbidity. Thus, we cannot determine whether NAC improved resectability or introduced delays. Also, subgroup findings should be interpreted cautiously, as multiple comparisons increase the risk of false positives. Additionally, although we excluded patients with synchronous or prior malignancies, the SEER database does not allow for accurate identification of subsequent primary malignancies diagnosed after gastrectomy. As such, some patients who developed a second cancer during follow-up may have remained in the cohort, potentially influencing overall survival estimates. Moreover, the absence of molecular data (e.g., MSI, EBV, Claudin-18.2, RHOA mutations) restricts our ability to define biological subgroups that may benefit from targeted or immunotherapy approaches [16,18,21,27,28].

Our results argue against universal NAC use in GSRCC. Instead, clinicians should consider patient and tumor characteristics—particularly location and stage—when planning treatment. The PRODIGE 19 trial will provide randomized evidence in this space [13], but until then, our findings suggest that NAC may benefit distal, locally advanced tumors while offering little value in early-stage or proximal disease.

Lastly, we observed racial disparities in outcomes, with Black patients experiencing worse survival despite adjustments. This highlights the importance of addressing systemic inequities in access to care, follow-up, and early detection efforts.

In summary, NAC should be considered selectively for GSRCC patients, particularly those with distal tumors or stage II/III disease. A one-size-fits-all strategy appears ineffective. Future research integrating molecular profiling, treatment response, and SRC quantification will be essential for advancing personalized approaches in GSRCC.

## 5. Conclusions

In this large SEER-based study of gastric signet-ring cell carcinoma, neoadjuvant chemotherapy did not significantly improve overall survival for the general population of patients. GSRCC remains a high-risk disease with poor outcomes under current treatment paradigms. However, our analysis indicates that a subset of patients (those with tumors in the mid/distal stomach) may experience improved survival with NAC. This finding suggests that the efficacy of NAC in GSRCC is context-dependent, and a tailored approach is warranted.

Our study underscores the urgent need for histology-specific clinical trials (like PRODIGE 19) and novel therapeutic approaches in GSRCC. In the meantime, clinicians managing GSRCC should weigh tumor location, stage, and patient factors when deciding on NAC. Multidisciplinary evaluation is critical, and patients should be counseled on the unclear benefit of NAC outside of potential subgroups. With further research and a precision oncology approach, we hope to improve the outlook for patients with this aggressive gastric cancer subtype.

## Figures and Tables

**Table 1:** Baseline clinicopathologic characteristics of SEER patients with gastric signet-ring cell carcinoma (2011–2018, *n* = 978).

Characteristic	Category	SEER (*n* = 978)
Gender	Male	517 (52.9%)
	Female	461 (47.1%)
Age at diagnosis	≤60 years	500 (51.1%)
	>60 years	478 (48.9%)
Race	Black	130 (13.3%)
	White	640 (65.4%)
	Asian (Chinese)	37 (3.8%)
	Other/unspecified	171 (17.5%)
Tumor location *	Proximal (cardia/fundus)	174 (17.8%)
	Mid (corpus/antrum) **	278 (28.4%)
	Distal (antrum/pylorus)	290 (29.7%)
	Overlapping regions ***	91 (9.3%)
	Unknown	63 (6.4%)
Tumor size	≤5 cm	450 (46.0%)
	>5 cm	248 (25.4%)
	Unknown	281 (28.7%)
Year of surgery	2011–2014	503 (51.4%)
	2015–2018	475 (48.6%)
Clinical TNM stage	II	202 (20.7%)
	III	776 (79.3%)

* Tumor location categorized based on primary site codes. ** Includes tumors involving both corpus and antrum regions. *** Overlapping indicates lesions spanning more than one region. Percentages may not total 100% due to rounding.

**Table 2:** Distribution of treatment modalities in the SEER cohort (*n* = 978).

Treatment Variable	Category	SEER (*n* = 978)
Neoadjuvant therapy	Received chemotherapy (NAC)	436 (44.6%)
	None (surgery first)	542 (55.4%)
Adjuvant therapy	Chemotherapy (post-op)	163 (16.7%)
	Chemoradiotherapy (post-op)	214 (21.9%)
	Radiotherapy only (post-op)	30 (3.0%)
	None	571 (58.4%)

**Table 3:** Pathologic characteristics of patients in the SEER cohort (*n* = 978).

Pathologic Variable	Category	SEER (*n* = 978)
Pathologic T stage	T1 (mucosa/submucosa)	277 (28.3%)
	T2 (muscularis propria)	164 (16.8%)
	T3 (subserosa/adjacent)	271 (27.7%)
	T4 (serosa or adjacent structures)	266 (27.2%)
Pathologic N stage	N0 (0 positive nodes)	395 (40.4%)
	N1 (1–2 positive nodes)	236 (24.1%)
	N2 (3–6 positive nodes)	185 (18.9%)
	N3 (≥7 positive nodes)	162 (16.6%)
Lymph nodes examined	Mean ± SD	27.2 (±39.7)
Lymph nodes positive	Mean ± SD	18.7 (±23.4)

Pathologic T and N stages are based on AJCC 7th edition criteria (as used during 2011–2018). Percentages for T and N may not total 100% due to rounding.

**Table 4:** Univariable and multivariable Cox regression analysis of factors influencing overall survival in SEER GSRCC patients.

Variable (Reference)	Univariable HR (95% CI)	*p*	Multivariable HR (95% CI)	*p*
Sex (Male ref)				
Female vs. Male	1.07 (0.91–1.26)	0.397	1.09 (0.91–1.30)	0.334 ^†^
Age (≤60 ref)				
>60 vs. ≤60	1.03 (0.87–1.22)	0.723	1.05 (0.88–1.26)	0.605 ^†^
Race (Black ref)				
White vs. Black	0.92 (0.72–1.18)	0.505	0.95 (0.74–1.22)	0.695
Asian (Chinese) vs. Black	0.56 (0.34–0.91)	0.019	0.58 (0.35–0.96)	0.033
Tumor size (≤5 cm ref)				
>5 cm vs. ≤5 cm	2.57 (2.14–3.09)	<0.001	1.67 (1.37–2.04)	<0.001
Tumor location (Proximal ref)				
Mid vs. Proximal	1.11 (0.89–1.37)	0.346	1.06 (0.85–1.33)	0.589
Distal vs. Proximal	1.14 (0.91–1.42)	0.265	1.12 (0.89–1.42)	0.318
Overlapping vs. Proximal	1.12 (0.83–1.50)	0.457	1.08 (0.79–1.48)	0.625
Clinical TNM stage (II ref)				
Stage III vs. II	3.38 (2.03–5.62)	<0.001	2.98 (1.59–5.58)	0.001
Pathologic T stage (T2 ref)				
T3 vs. T2	2.74 (2.14–3.52)	<0.001	1.52 (1.08–2.15)	0.017
T4 vs. T2	4.61 (3.54–6.01)	<0.001	2.13 (1.45–3.13)	<0.001
Pathologic N stage (N0 ref)				
N1 vs. N0	1.85 (1.45–2.37)	<0.001	1.34 (1.02–1.77)	0.035
N2 vs. N0	2.01 (1.53–2.64)	<0.001	1.25 (0.89–1.75)	0.197
N3 vs. N0	3.09 (2.40–3.97)	<0.001	1.64 (1.20–2.25)	0.002
Year of surgery (2011–14 ref)				
2015–2018 vs. 2011–2014	0.93 (0.78–1.11)	0.419	0.89 (0.74–1.07)	0.229
Neoadjuvant therapy (NAC vs. none)				
No NAC vs. NAC	0.94 (0.77–1.15)	0.557	0.91 (0.74–1.13)	0.397
Adjuvant therapy (Yes ref)				
No adjuvant vs. Yes	1.08 (0.81–1.43)	0.610	1.17 (0.88–1.57)	0.285

^†^ Not statistically significant and excluded from the multivariable model based on backward stepwise selection or due to collinearity. Reference categories for each variable are indicated. HR: hazard ratio; CI: confidence interval; ref: reference group; NAC: neoadjuvant chemotherapy.

## Data Availability

Publicly available datasets were analyzed in this study. These data can be found in the Surveillance, Epidemiology, and End Results (SEER) database maintained by the U.S. National Cancer Institute: https://seer.cancer.gov/.

## Abbreviations

The following abbreviations are used in this manuscript:

GSRCC	Gastric Signet-Ring Cell Carcinoma
SEER	Surveillance, Epidemiology, and End Results
NAC	Neoadjuvant Chemotherapy
OS	Linear dichroism
HR	Hazard Ratio
CI	Confidence Interval
TNM	Tumor–Node–Metastasis (staging system)
KM	Kaplan–Meier
